# Effect of behavioral interventions on contraceptive use among women living with HIV: Systematic review and meta-analysis of randomized controlled trials

**DOI:** 10.1186/s12978-026-02391-0

**Published:** 2026-06-24

**Authors:** Michael Deynu, Jerry John Ouner, Sarah A. Gutin, Ifeyinwa V. Asiodu, Peggy Tahir, Glenn-Milo Santos

**Affiliations:** 1 Department of Family Health Care Nursing, School of Nursing, University of California San Francisco, San Francisco, CA 94158 USA; 2 Department of Community Health Systems, School of Nursing, University of California San Francisco, San Francisco, CA 94158 USA; 3 University of California San Francisco Library, University of California San Francisco, San Fracisco, CA 94143 USA

**Keywords:** Behavioral interventions, Counseling, Contraception, Systematic review, Meta-analysis, Unplanned pregnancy, HIV

## Abstract

**Background:**

Contraceptive use is a cost-effective public health strategy to prevent unplanned pregnancy, maternal mortality and morbidity, and reduce vertical HIV transmission. However, contraceptive use remains low among women living with HIV. Reviews examining effect of behavioral interventions on contraceptive use among women living with HIV using randomized controlled trials are limited. We evaluated the effect of behavioral interventions on contraceptive use among women living with HIV using randomized controlled trials.

**Methods:**

We conducted a systematic literature search across PubMed, Embase, Web of Science, Cumulative Index to Nursing and Allied Health Literature, ClinicalTrials.gov and the Cochrane Central Register of Control Trials from their inception to 09/15/2025. The Preferred Reporting Items for Systematic Reviews and Meta-Analysis (PRISMA-2020) guidelines were followed in conducting this review. Peer reviewed and published randomized controlled trials (RCTs) focusing on behavioral interventions were included. Effect estimates (odds ratios) were pooled using a random-effects model (REM). Quality of the evidence was assessed using the Grading of Recommendations, Assessment, Development and Evaluation (GRADE) approach.

**Results:**

The search yielded 853 records. Five trials involving 5903 women living with HIV and published between 2009 and 2021 were included in this review. Across the trials, structured contraceptive counseling was the most common intervention implemented while others included provision of family planning vouchers, client outreaches and education and counseling on safe sex, and social support. Two of the studies were conducted in Uganda, two in Kenya and one in the United States. Two of the studies reported 100% antiretroviral therapy use with mean duration ranging 4.1 years (SD = 3.33) to 7.8 years (SD = 7.1), while one study reported 83.9% viral suppression among their sample. Across the trials, behavioral interventions were significantly associated with greater odds of contraceptive use among women living with HIV with pooled odds ratio, 3.61 (95% CI: 1.15, 11.32), *p =* 0.04 compared to controls.

**Conclusions:**

The findings underscore the efficacy of behavioral interventions in enhancing contraceptive use among women living with HIV. Leveraging behavioral interventions could offer an opportunity to increase contraceptive use and improve the reproductive health outcomes for women living with HIV.

**Supplementary Information:**

The online version contains supplementary material available at https://doi.org/10.1186/s12978-026-02391-0.

## Abstract (plain language summary)

Women living with experience high unplanned pregnancy and contraceptive discontinuation compared to women without HIV. Among women living with HIV, an estimated 60%-70% of pregnancies are unplanned; and are often associated with preterm birth, small for gestational age babies, low birth weight and stillbirth. Contraceptive use is an important public health strategy to prevent unplanned pregnancy, lowers the risk of mothers dying, and reducing the risk of passing HIV from the mother to the unborn child. However, many women living with HIV face significant barriers to contraceptive use, although they experience high unplanned pregnancy. Ensuring that these women have access to family planning methods can help prevent unplanned pregnancies and improve their reproductive health outcomes. Further, despite the benefits of family planning in preventing unplanned pregnancy and HIV transmission from the mother-to-the-child, many women living with HIV lack access to or do not utilize contraceptives. Studies examining the effect of behavioral interventions on contraceptive use among women living with HIV are currently lacking. Therefore, we examined the effect of behavioral change interventions in enhancing contraceptive use among women living with HIV.

Several databases were searched and randomized controlled trials that examined the effect of behavioral interventions on contraceptive use among women living with HIV were included. The search yielded 853 records. Five trials involving 5903 women living with HIV were included. Across the five trials, contraceptive counseling was the most common intervention. Two trials were conducted in Uganda, two in Kenya and one in the United States. Two of the studies reported 100% anti-retroviral therapy use while one study reported 83.9% viral suppression. In each of the trials, contraceptive use increased among women who received the intervention compared to those who did not. Across the studies, behavioral interventions demonstrated significant effect in improving contraceptive use among women living with HIV. The findings show that behavioral interventions may have the potential to improve contraceptive use among women living with HIV. Optimizing these interventions could help enhance contraceptive use, reduce unplanned pregnancy and prevent HIV infection from the mother to the child among women living with HIV.

**Supplementary Information:**

The online version contains supplementary material available at https://doi.org/10.1186/s12978-026-02391-0.

## Introduction

Globally, HIV remains a global public health issue with women and girls accounting for nearly 44% of all new HIV infections and 53% of the total HIV burden [1, 2]. Antiretroviral therapy (ART) use and HIV status influence fertility desires among women living with HIV (WLHIV) [3–5]. Positive HIV status negatively influence child bearing desires, often compounded by societal disapproval for childbirth, concerns that WLHIV may not live long, vertical HIV transmission, and perceived negative impact on their overall health [6]. Pregnancy desire varies with some studies estimating 40%−55% of WLHIV expressed their desire not to become pregnant [7, 8], while 42%−48% indicated pregnancy desire [9, 10]. Despite high unplanned pregnancy among WLHIV, contraceptive use is estimated between 25.1% and 30.1% in some study reports [11, 12]. Notably, 60%−70% of pregnancies among WLHIV are unplanned [13, 14], although this may be lower in parts of Africa [15]. Unplanned pregnancies are often associated with adverse perinatal and maternal outcomes, including preterm birth, small for gestational age babies, low birth weight and stillbirth [16, 17], Further, women living with untreated HIV infection are more likely to transmit HIV to their newborn infants which may be due to non-use or non-adherence to antiretroviral therapy (ART) [18] and unsafe sexual practices [19, 20].

Modern contraceptives are cost effective public health interventions that can prevent unplanned pregnancy and reduce the risk of vertical HIV transmission [21, 22]. Beyond its potential in preventing unplanned pregnancy among women with maternal HIV infection, contraception reduce rates of complications associated with neonatal mortalities and unsafe pregnancy terminations [23–25], thus increasing quality of life and general well-being [26]. Despite these benefits and availability of a wide range of contraceptive methods, contraceptive use has been suboptimal and varied among WLHIV in Sub-Saharan Africa (SSA) [27–30], including parts of the global North [31, 32]. The low contraceptive use is influenced by fertility desires, HIV status disclosures, marital status, nature and quality of contraceptive counseling, educational attainment, partner involvement in post-test counseling and highly active antiretroviral therapy use, and sero-discordant states [19, 27–29, 33]. Further, unprotected sexual practices have been noted among women living with HIV due to challenges associated with negotiating contraception, domestic violence, alcohol use and reproductive intentions [34, 35]. Behavioral interventions including high quality, individual counseling and support services, integration of contraceptive services into HIV care, use of peer navigators living with HIV, and guided reproductive health education have all shown significant promise in addressing barriers associated with low contraceptive use among women living with HIV [36–38].

In the current literature however, limited prior reviews have evaluated the influence of intervention strategies in improving contraceptive use among women living with HIV [39–41] and adults living with HIV [42]. These interventions generally included counseling, use of mobile phones for reminders and educational materials tailored towards individuals and groups [39]. Although findings from these reviews remain valid, they are limited in many ways. For instance, these reviews included non-randomized studies which limited statistical pooling of effect estimates [39], focused primarily on condoms while excluding other modern contraceptive methods [40, 41], and the adult population living HIV [43]. While there is a limited body of literature on the effect of behavior change interventions in improving contraceptive use [44, 45], findings from these reviews are also heterogeneous, due to diverse study designs, interventions and study populations [39, 40, 43, 46]. Altogether, information on the effect of behavioral interventions on contraceptive use outcomes using randomized controlled trials among women living with HIV remains unclear and currently lacking. This may have significant implications for reproductive health programming within HIV care and warrants reviews that provide evidence to guide public health efforts aimed at improving contraception, reduce unplanned pregnancy and enhance reproductive health outcomes for women living with HIV.

### Purpose of the review

Given the high rates of unplanned pregnancy among WLHIV and the critical role contraception plays in reducing unplanned pregnancy and improving maternal and child health outcomes among this population, there is the need for current reviews that quantitatively evaluate the effect of behavioral interventions using randomized controlled trials to better understand their impact on contraceptive use outcomes among WLHIV. Similarly, the limitations of prior reviews warrants new studies that provide evidence that can help inform evidenced-based reproductive health programming and policy reforms within HIV care that is aimed at enhancing contraceptive use, reduce unplanned pregnancy and improve health outcomes for WLHIV. Because factors influencing contraceptive use among WLHIV are likely to be complex [47, 48], addressing these gaps could help improve HIV care and family planning programming and identify effective interventions that could enhance contraceptive use, reduce unplanned pregnancy and improve health outcomes for women living with HIV. This systematic review and meta-analysis therefore, examined the effect of behavioral interventions in improving contraceptive use among women living with HIV using randomized controlled trials.

### Review question

Among women living with HIV, does behavioral interventions compared to usual care improve contraceptive use?

## Methods

This systematic review and meta-analysis has been registered in the International Prospective Register of Systematic Reviews (PROSPERO) with the specific registration number CRD42025639444.

### Reporting

The Preferred Reporting Items for Systematic Reviews and Meta-Analysis (PRISMA 2020) guidelines [49] were followed in the conduct of this review (Supplementary File 1).

### Search strategy

We systematically searched five electronic databases including PubMed (January 1996), Embase (May 1974), Web of Science (January 1997), and Cumulative Index to Nursing and Allied Health Literature (CINAHL) (1961) from their inception to 09/15/2025. The search strategy was developed in partnership with an expert medical librarian using Medical Subjects Heading (MESH) indexing terms and keywords. Additional searches were also conducted in the Cochrane Central Register of Controlled Trials (1996) and ClinicalTrials.gov (February 2000) from their inception to 09/15/2025. In addition, complimentary searches were conducted through citation chaining and hand searching. Concepts were searched using keywords and Boolean operators “OR” and “AND”. No date limitation was applied to the literature search.

The example search terms and key words used were;

(“family planning” OR “family planning services” OR "contraception” OR “contraceptives” OR “contraceptive” OR “contraceptive agents” OR “contraceptive devices” OR “intra-uterine device” OR "intrauterine device" OR “intra-uterine devices” OR “intrauterine devices” OR “birth control” OR “birth spacing” OR “birth intervals” OR “planned pregnancy” OR “planned pregnancies”) AND (“Human Immunodeficiency Virus” OR "HIV" OR “Immunodeficiency Virus” OR “Acquired Immune Deficiency Syndrome” OR “AIDS” OR “women living with HIV” OR “HIV seropositive” OR “HIV Seropositivity” OR “infected with HIV” OR “HIV infection”) AND (“behavioral interventions” OR “counseling” OR “contraceptive counseling” OR “reproductive counseling” OR “sex counseling” OR “psychotherapy” OR “psychosocial interventions” OR “motivational interviewing” OR "community-based family planning" OR “family planning vouchers” OR “vouchers” OR “texting” OR “text messaging” OR “mHealth” OR “eHealth” OR “telehealth” OR “tele-health” OR “tele-counseling” OR “tele counseling” OR “cash transfers” OR “conditional cash transfers”) AND ("randomized controlled trial" “RCT” OR “randomized trial”). The full search strategy for the various databases is attached in Appendix 1.

### Inclusion and exclusion criteria

To be eligible for inclusion, studies were to be 1) conducted among women living with HIV aged 15–49 years, 2) randomized controlled trials and 3) focused onbehavioral interventions that are related to increasing contraceptive use, 4) reported on modern contraceptive use, 5) peer reviewed articles and published in English language. Further, multi-component intervention studies were considered eligible when the intervention has at least one behavioral component. Studies conducted on men living with HIV were excluded. Further, randomized controlled trials that do not report data on contraceptive use as an outcome measure were excluded. The literature search was limited to citations published in English language only. Table 1 below details the full eligibility criteria.

**Table 1:** Inclusion and exclusion criteria

Study characteristics	Inclusion criteria
Population	Women aged 15–49 years living with HIV
Interventions	Contraceptive counseling, psychotherapy, motivational interviewing, community-based family planning, family planning vouchers, text messaging, cash transfers, mHealth, and telehealth interventions that are related to increasing contraceptive use
Comparator	Informational pamphlets and videos, general health education, regular clinic visits or usual care
Outcomes	Contraceptive use of any modern method (pills, male and female condoms, injectables, intra-uterine devices, contraceptive implants, lactational amenorrhea method (LAM), male and female sterilization
Study design	Randomized controlled trials
Reporting Characteristics	
Language of publication	Abstracts and full texts published in English language
Publication status	Peer reviewed and published studies
Study settings	Community and institutional based studies No limitation to country

### Study selection

Records retrieved from databases were exported to Endnote 20.0, and then to Covidence where duplicate studies were removed. The title and abstracts of the remaining citations were independently screened by MD and JJO against the eligibility criteria for inclusion into full text review. Disagreements arising regarding an article inclusion into full text review were resolved through consensus and discussions. A third reviewer, GMS adjudicated any disagreements that remained unresolved. The Cohen’s Kappa (a statistical measure of the agreement between two raters) [50] score was 0.52 while the proportionate agreement (a statistical value denoting agreement/disagreement between two raters) [50] was 0.92.

### Data extraction

MD and JJO independently extracted data from the included articles using an Excel data extraction form designed a priori to ensure systematic data representation from each of the included studies. Relevant data on author(s), year of publication, study population, setting, sample size, participant age, study design, type of intervention, dose and frequency of intervention were extracted from each of the included studies. Data on follow-up duration and outcome assessment methods were extracted. Further, data was also extracted on effect sizes (odds ratio) and 95% confidence intervals, proportion of contraceptive use and theoretical frameworks from each of the studies included.

### Outcome data

The main outcome in this review is contraceptive use among women living with HIV. This was defined as a binary outcome (yes/no). Contraceptive use was defined as the practice and use of medications, product or surgical procedures to prevent unplanned pregnancy and to attain a desired number of children [51, 52]. These include oral contraceptive pills (OCPs), male and female condoms, injectables, emergency contraceptive pills (ECs), subdermal implants, intra-uterine devices, male and female sterilization, and lactational amenorrhea methods (LAM) [51, 52]. Similar contraceptive methods were reported in majority of the studies. When studies report multiple outcome time points, we selected the effect estimate closest to the commonly reported time period across the studies to maintain comparability.

### Risk of bias assessment

MD and JJO independently assessed risk of bias and methodological quality of included studies. Disagreements were resolved through discussion, and where unsuccessfully resolved, a third reviewer, GMS adjudicated. The risk of bias and methodological quality were assessed using the revised Cochrane Risk of bias tool for randomized controlled trials (RoB 2.0) [53, 54]. The Cochrane Risk of bias tool addressed the following domains: 1) bias regarding the randomization process, 2) bias due to deviations from the intended interventions, 3) bias due to missing outcome data, 4) bias in outcome measurements and 5) biases related to selection of reported results [53]. Overall, studies were classified as “some concerns” if a study had some concerns judgement across multiple domains and low risk if all domains were low risk. Results of methodological quality and risk of bias assessment were presented graphically using the web-based robvis (RoB 2.0) tool [53].

### Assessment of quality of evidence

We assessed quality of the evidence using the Grading of Recommendations, Assessment, Development and Evaluation (GRADE) approach [55, 56]. The GRADE’s approach to rating evidence quality begins with the study design and then addresses five domains: risk of bias (major methodological limitations-lack of randomization, allocation concealment or blinding), imprecision (risk of random error), inconsistency (systematic differences across study results), indirectness (applicability, generalizability, external validity and transferability) and publication bias to downgrade or upgrade the quality of the evidence [56]. The quality of the evidence were lowered due to concerns regarding risk of bias in a study, any inconsistencies in study findings, concerns regarding indirectness, overall data precision or evidence that suggests publication bias. Further, the rating could be upgraded if a study demonstrated large effect, dose–response relationship, and considered residual confounding which may have influenced study outcomes. The summary of findings (SoF) table was generated using the Cochrane GRADEpro GDT [57].

### Data synthesis

We extracted relevant data in Microsoft Excel format and all analysis were conducted using the *metan* command in STATA version 18.0 [58]. Odds ratios (OR) extracted from the included studies were used to generate pooled effect estimates irrespective of the quality of the studies. We conducted a random-effects modelling (REM) using a restricted maximum likelihood (REML) approach to estimate the weighted summary effect estimate and 95% Confidence Interval (CI) of the efficacy of behavioral interventions [59–62]. Individual study weights were calculated as the inverse of the sum of within-study variance and between study variance which accounted for both sampling error and heterogeneity across the studies [59, 60]. We estimated confidence intervals for the pooled effect estimate using the Hartung-Knapp-Sidik-Jonkman (HKSJ) approach [62]. The random-effects model (REM) was adopted because we hypothesized that the true intervention effect estimates were heterogenous across the included studies and were estimated to follow a normal distribution [59, 60]. We calculated 95% prediction interval (PI) for the primary analysis, (k = 5) to estimate the range within which the true effect estimate of a future study is expected to lie [62]. Heterogeneity was assessed using the Cochrane Q statistics and the *I*^2^ statistics [63, 64].

A Q-statistics, *p <* 0.05 were indicative of heterogeneity. The *I*^2^ statistic was used to estimate the proportion of heterogeneity in the effect estimate of the included studies that were attributable to methodological or clinical variabilities [61, 63–65]. An *I*^2^ statistic ≤ 40%, between 30% ≤ *I*^2^ ≤ 60%, 50% ≤ *I*^2^ ≤ 90%, and > 75% were interpreted as not significant, mild, moderate and high heterogeneity, respectively [63, 64]. The summary effect estimates and 95% confidence intervals (CI) of the individual study outcomes were represented using forest plots [66–68]. To investigate the sources of heterogeneity, subgroup analyses were conducted and restricted to results of the risk of bias and methodological quality assessments; low-risk bias studies, study setting (those conducted in Africa) and the most common intervention type (contraceptive counseling). In addition, we conducted meta-regression, considering intervention type, study setting and study quality to explore the sources of heterogeneity. These covariates were selected based on our hypothesis that they could modify intervention effects and contribute to between-study heterogeneity. We calculated absolute risk in Cochrane GRADEpro GDT. The assumed risk in the usual care arm was derived from event rates of contraceptive use and represents the proportion of study participants reporting contraceptive use at follow-up.

### Sensitivity analysis

We conducted sensitivity analysis to assess the influence of individual study effect estimates on the pooled effect size using the “leaveoneout” (LOO) approach in STATA. This involved iteratively removing one study at a time and recalculating the pooled effect to determine the effect of individual studies on the pooled effect. A study was considered influential if its exclusion materially alter the magnitude or direction of the pooled effect estimate.

## Results

### Search results

**Fig. 1:**
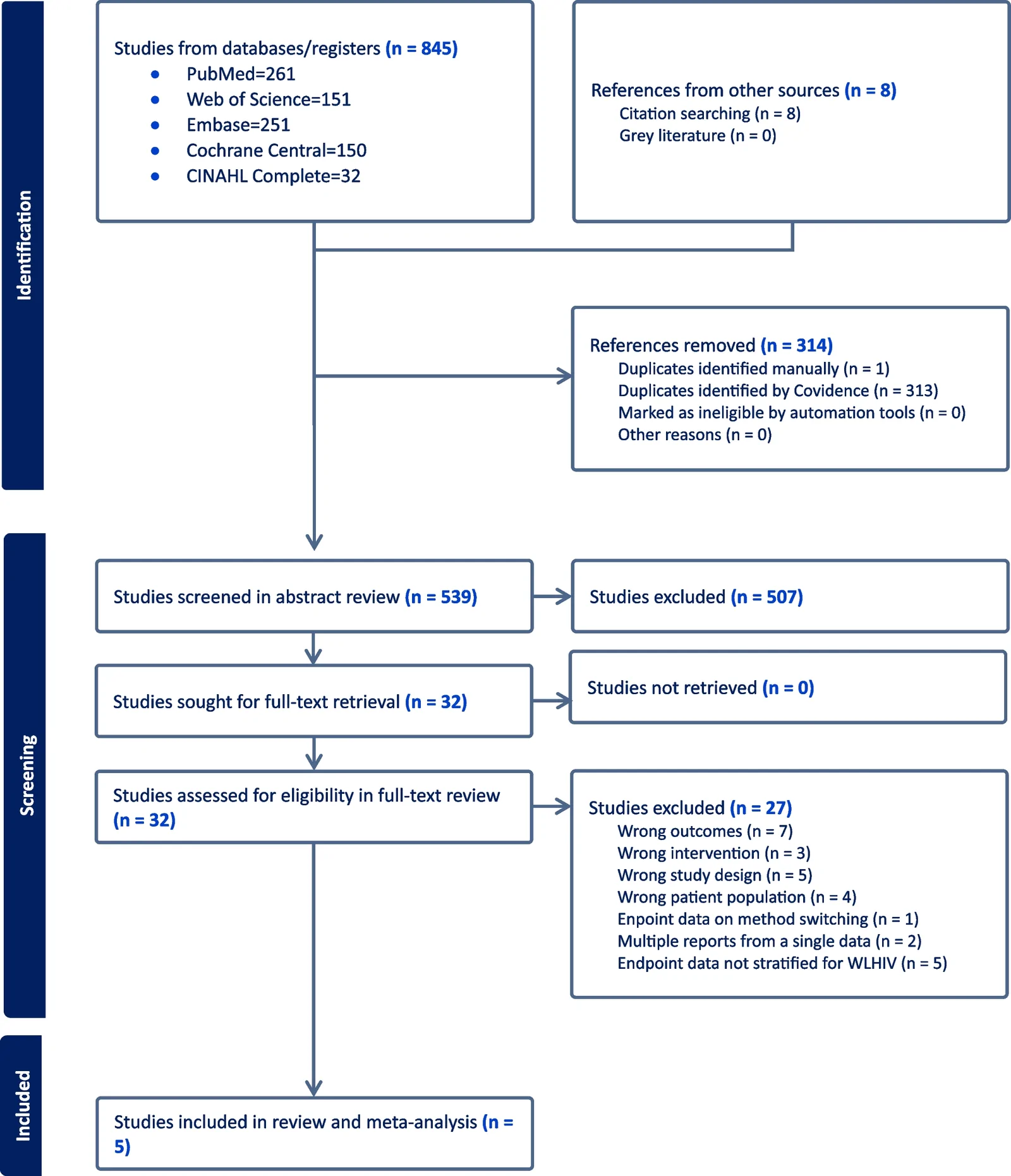
PRISMA Flow diagram of study selection and inclusion

The PRISMA diagram below summarizes the identification, screening and inclusion of studies in this review (Fig. 1). A total of 853 studies were identified. Out of these, 314 duplicate studies were removed after which 539 articles were screened. Following this, 507 articles that did not meet the inclusion criteria were excluded and 32 studies were then selected for full text review.

The 32 full text citations were then retrieved and reviewed, out of which 27 potentially relevant articles were excluded for various design and outcome related issues. These studies were excluded for not meeting the criteria for inclusion based on: outcomes [69–75], intervention type [76–78], study design [79–83], patient population [84–87], contraceptive method switching [88], and outcome data not stratified for women living with HIV [89–92]. Further, two studies each were excluded because they reported data from two other studies that were already included [93, 94]. This was done to avoid double counting. Five (5) trials that ultimately met the inclusion criteria were included in this review.

### Description of studies included

A total of five (5) randomized controlled trials enrolling 5903 women living with HIV were included [95–99]. Two of the studies were conducted in Uganda [95, 96], two in Kenya [97, 99] and one in the United States of America (USA) [98]. Three of the studies were conducted and published between 2010 and 2019 [95–97] while two were conducted between 2004 and 2008 [98, 99]. Across the studies, age ranged 30 years to 40 years [95–99]. Two of the studies in Uganda implemented structured counseling on contraceptive methods, method choices, benefits and side effects, client outreaches, provision of contraceptives and family planning vouchers [95, 96]. One study conducted in Kenya integrated family planning counseling and provision of family planning methods at HIV clinics [97], while the trial conducted in the United States offered group level intervention focusing on counseling on safe sex, risk reduction and skills building and social support [98]. Another study in Kenya issued contraceptive appointment cards, male partner involvement during counseling in addition to training of clinical and counseling staff on contraceptive methods, practical demonstrations and discussions on myths and barriers and provision of free contraceptive methods [99].

Across the five studies, two underpinned their intervention development and implementation with the Information, Motivation and Behavioral Skills (IMB) Model [96] and the Transtheoretical Model of the Stages of Change, Modified AIDS Risk Reduction Model, Theory of Gender and Power [98], respectively. The sample sizes ranged from 159–3584 [95–99]. Contraceptive use was assessed using self-reports across the five studies included [95–99]. Two studies reported all women were currently using anti-retroviral therapy with a mean ART duration ranging from 4.1 years (SD = 3.33) to 7.8 years (SD = 7.1) [95, 96]. One study reported 83.9% viral suppression among their sample [96]. The detailed characteristics of the studies included in the systematic review and meta-analysis are presented in Table 2 below.

**Table 2:** Characteristics of studies included in the systematic review and meta-analysis

Study ID & Pub.Year	**Country & Setting**	**Study Year**	**Population**	**Mean Age (years)**	**Sample size**	**Design**	**Type of intervention and components**	**Controls**	**Dose & frequency of Intervention**	**Intervention Effec**t	**Theoretical frameworks**
Atukunda et al. 2021 [95]	Uganda	Oct 2016-June 2018	Adult women living with HIV	Intervention group, mean age 30.0, (SD = 5.9), Control group, 29.6(6.0)	317	Randomized controlled trial	Structured counseling on available contraceptive methods, family size choices and desires, when to start, how to use methods, benefits and potential side effects, and where to access family planning methods Counseling on standard days method (SDM) and Lactational Amenorrhea Method (LAM) Provision of family planning vouchers as an incentive and offers an opportunity for free administration (injection, intra-uterine device, implant) and daily SMS reminders for adherence support	Routine family planning counseling	Structured counseling lasting 40 min Daily SMS reminders for first 4 months and then weekly for the next 2 months	Contraceptive use was 79.8% in the intervention group compared to control (69.2%) aOR = 1.75[1.24–2.75]	Not reported
Wagner et al. 2021 [96]	Uganda	July 2017—Jan 2019	Adult women living with HIV(sero-discordant)	Overall, mean age, 35.9(SD = 8.2) Intervention group, 35.1(7.2) Control group 37.1(9.1)	389	Randomized controlled trial	Counselling to facilitate an informed childbearing decision-making and support on contraception, client outreaches to increase contraceptive awareness and uptake, provision of safer conception counseling, routine screening for childbearing desires and provision of contraception, discussions on risk reduction	Usual provision of family planning, no training provided	Initial consultation lasting 45–60 min Safer conception counseling lasting 20 30 min Follow up sessions lasting 20 min	Appropriate reproductive method use was higher in the intervention (20.8%) compared to control (6.9%) aOR = 10.63[2.79,40.49]	Information, Motivation and Behavioral Skills Model
Grossman et al. 2013 [97]	Kenya	Dec 2009 Sep 2011	Adult women living with HIV	Median age at intervention site 31.0, (IQR = 26–36) Control site, median age 30(IQR = 25–36)	3584	Randomized controlled trial	Integration of family planning counseling and provision of all reversible family planning methods and condoms at the HIV clinics	Standard practice of client referral to maternal and child health family planning clinic at the same facility, availability of family planning services at different times requiring women to return for family planning services later	Not Reported	Contraceptive use increased from 16.7% to 36.6% at intervention sites compared to 21.1 to 29.8% at control sites At 12 months, OR = 1.81[1.24,2.63]	Not reported
Teti et al. 2010 [98]	United States of America	April 2004—July 2006	Adult women living with HIV	Mean age at intervention group 40.7(SD = 8.3) Control arm, 38.9(SD = 8.8)	184	Randomized controlled trial	Group level counseling sessions focused on safe sex, risk reduction education and skills building, women challenges and opportunities, facts on HIV/AIDS and STIs, male and female condom use negotiation, goal setting, problem solving, triggers for safe sex, HIV status disclosures, and social support	Standard brief educational message by healthcare providers (HCPs) during routine medical visits	Five weekly sessions lasting 1 h 30 min delivered by health educator, and followed by weekly peer-led support groups	At baseline: contraceptive use was (intervention-67.4% versus 48.2%; 6 months-71.5% versus 49.5%; 12 months-52.2%−52.5% and 18 months- 74.1% versus 48.5%) at the control group At baseline, aOR = 4.58 [0.85, 24.74] At 6 months, aOR = 17.13[2.96, 99.10] At 12 months, aOR = 1.52[0.25,9.24] At 18 months, aOR = 270.04[24.53,2971.94]	Transtheoretical model of the Stages of Change, Modified AIDS Risk Reduction Model, Theory of Gender and Power
Ngure et al. 2009 [99]	Kenya	Dec 2004- Oct 2008	Adult women living with HIV	Not reported	1429	Randomized controlled trial	Provision of free contraceptive methods (oral contraceptive pills, injectables, implants, and intra-uterine devices Use of contraceptive appointment cards with dates for renewal of the time-dependent methods (injectable depot medroxyprogesterone acetate) to avoid lapses in hormonal contraception Designation of one staff to ensure adequate supply of contraceptives Introduction of checklists in chart notes to remind staff to discuss contraceptive methods Discussion of challenges to contraceptive uptake with clients individually and in psychosocial support groups Behavioral intervention focusing on male partner involvement during contraceptive counseling: couples family planning counseling Behavioral intervention focusing on training of clinical and counseling staff on contraceptive methods, including practical demonstrations, and discussion of common myths and barriers to contraceptive use	Referral of women to nearby medical facilities	Exact intervention dose and duration were not reported but there were weekly meetings with clinicians, counselors and pharmacy staffs to discuss contraception with participants	Contraceptive use from 31.5% to 64.7% after implementation of the intervention, aOR = 4.0[3.0,5.3] Contraceptive use changed from 15.6% to 22.3% in the control arm, aOR = 1.5[1.3,1.9]	Not reported

### Description of intervention

Across the studies, multi-component interventions were implemented (Table 2). The interventions were diverse and included individual and group counseling on family planning methods and use, family size choices, provision of family planning vouchers, safe sex, and condom use negotiation. Others included free provision of contraceptive methods, integration of counseling on family planning into HIV care, couple counseling, use of appointment cards for renewal of time-dependent contraceptive methods, checklists for reminding staffs to discuss contraceptive methods, and daily SMS. In addition, interventions also included, weekly meetings with clinicians to discuss user experiences, psychosocial support, male partner involvement during counseling, and client outreach to increase awareness and uptake of contraceptives. The duration, length and dose of interventions varied across the studies.

### Risk of bias and methodological quality of included studies

**Fig. 2:**
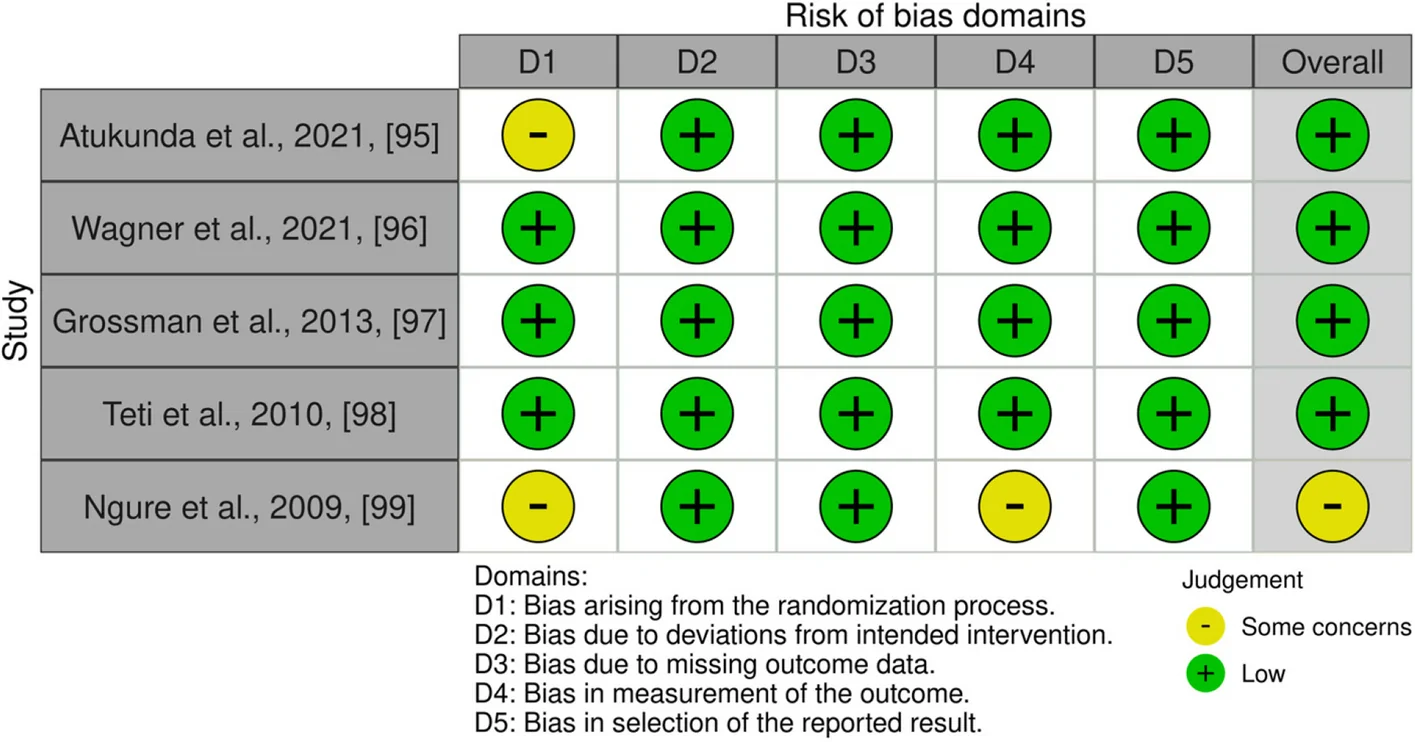
Risk of bias assessment

**Fig. 3:**
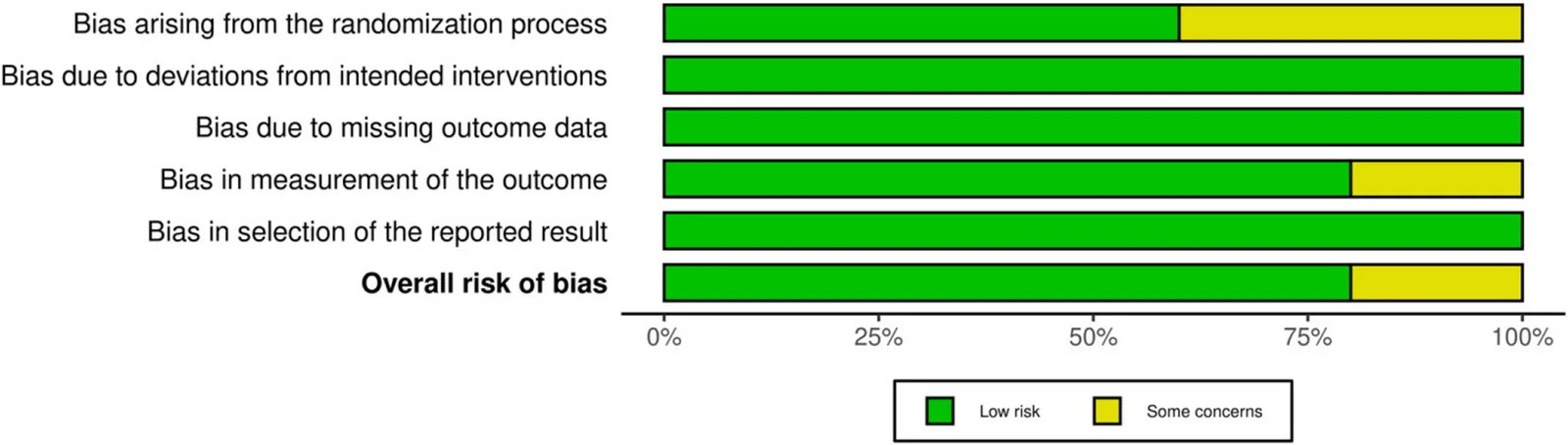
Risk of bias assessment

The Figs. 2 and 3 below presents results of the risk of bias and methodological quality assessment for each of the studies included across five domains; biases arising from randomization, deviations from planned interventions, missing outcome data, assessment of the outcomes and selection of the reported results [53]. Across the five (5) studies, four (4) were judged as low risk bias [95–98] and one was judged as a study with some concerns [99].

### Efficacy of behavioral interventions

**Fig. 4:**
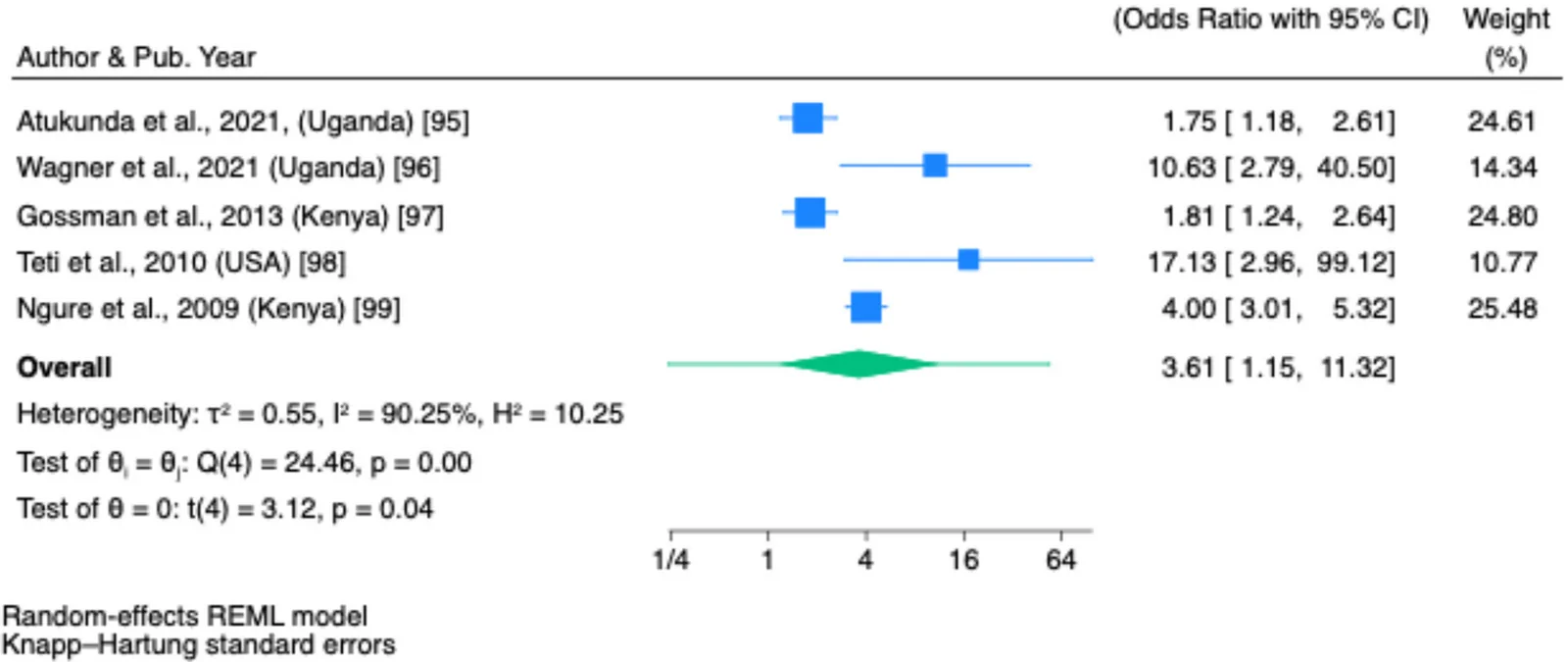
Forest plot of pooled effect estimate of behavioral interventions on contraceptive use

Five trials sampling 5903 women living with HIV reported effect estimates of behavioral interventions in increasing contraceptive use [95–99]. Across the trials, behavioral interventions were significantly associated with greater odds of contraception with a pooled odds ratio of 3.61 (95% CI: 1.15, 11.32), *p =* 0.04 (Fig. 4). Also, it is worthy to note that the point estimates of each of the included studies showed statistically significant positive effect of behavioral interventions on contraceptive use. We noted substantial heterogeneity with a Q-statistic, *p <* 0.001 and I^2^ index of 90.25%. The 95% prediction interval (PI) was (0.24, 53.74), suggesting that the true effect measure in future studies may lie within this range. The I^2^ showed that 90.25% of the total variation across the included studies could be due to heterogeneity [63, 64, 100]. This may plausibly be due to variations in intervention components, control conditions, intervention dose and frequency and how the interventions were designed and implemented.

### Subgroup analysis

**Fig. 5:**
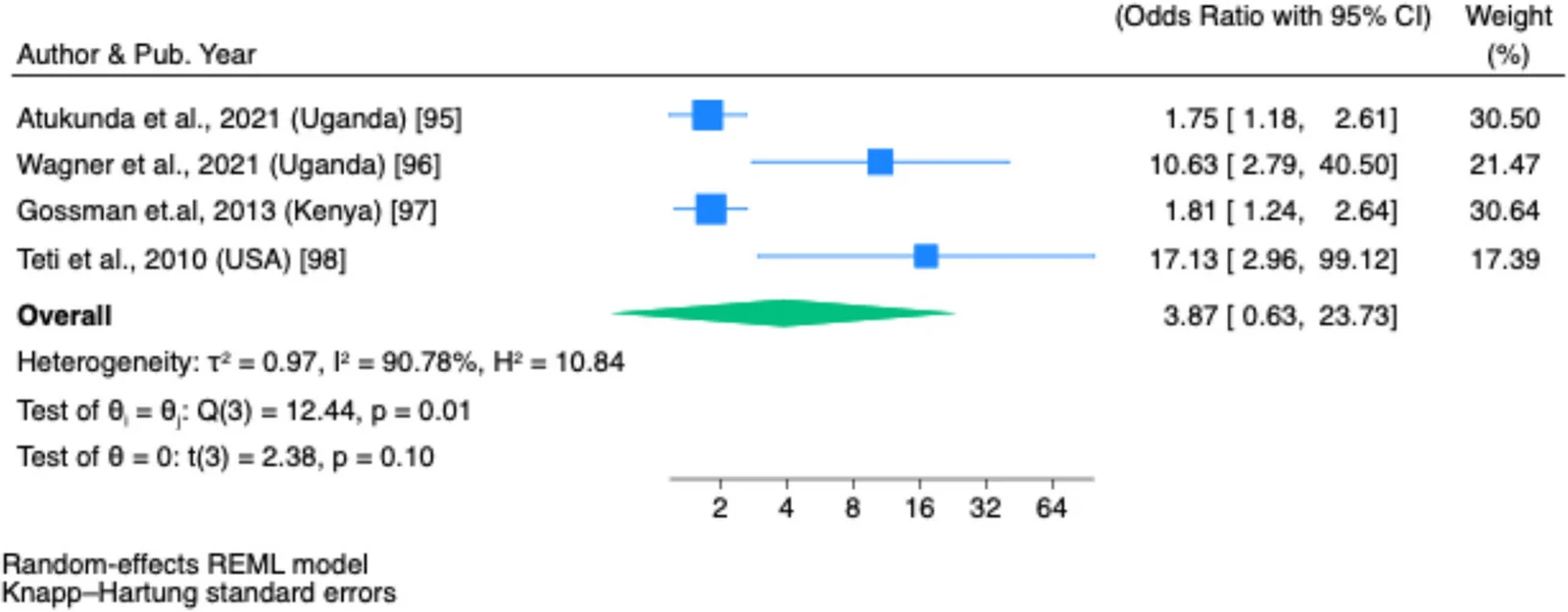
Forest plot of pooled effect estimate of behavioral interventions on contraceptive use in low bias studies

To examine the source of the high heterogeneity, subgroup analyses were conducted restricting studies to the following characteristics; low risk studies, type of intervention implemented (contraceptive counseling) and country or setting (in Africa) where trials were conducted. Across the studies judged as low-risk of bias [95–98], the pooled odds ratio was 3.87 (95% CI: 0.63, 23.73), *p =* 0.10 (Fig. 5). The I^2^ was 90.78%, *p =* 0.01. To investigate whether effect estimates differ by risk of bias, we conducted a random effects meta-regression. The test for subgroup differences was not statistically significant, *p =* 0.98. However, the test statistic may be underpowered due to the limited number of trials and should therefore be interpreted with caution.

**Fig. 6:**
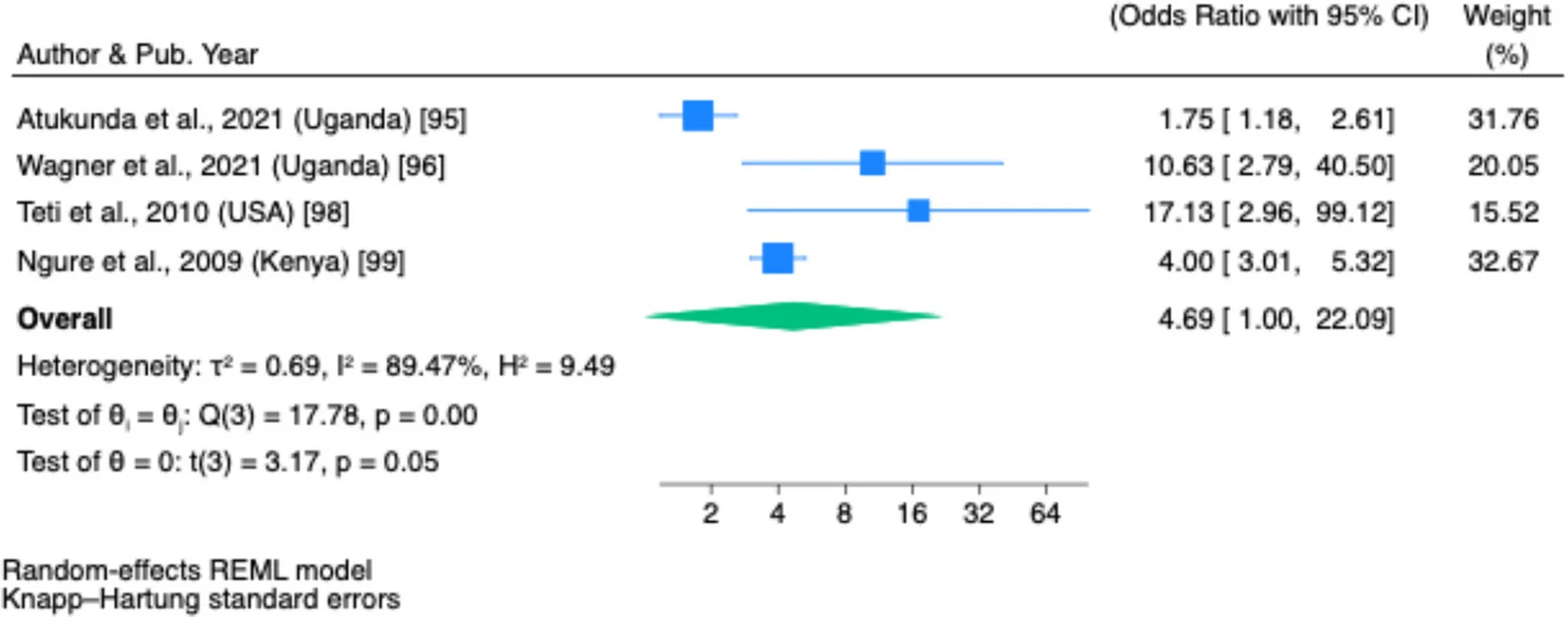
Forest plot of pooled effect estimate of behavioral interventions on contraceptive use in studies that implemented contraceptive counseling

In the subgroup analysis of trials that implemented contraceptive counseling as part of their intervention [95, 96, 98, 99], the pooled odds ratio was 4.69 (95% CI: 1.00, 22.09), *p =* 0.05 (Fig. 6). The I^2^ was 89.47%, *p <* 0.001, indicating variability between studies and uncertainty around the pooled estimate. The test for subgroup differences showed no statistically significant differences, *p =* 0.407.

**Fig. 7:**
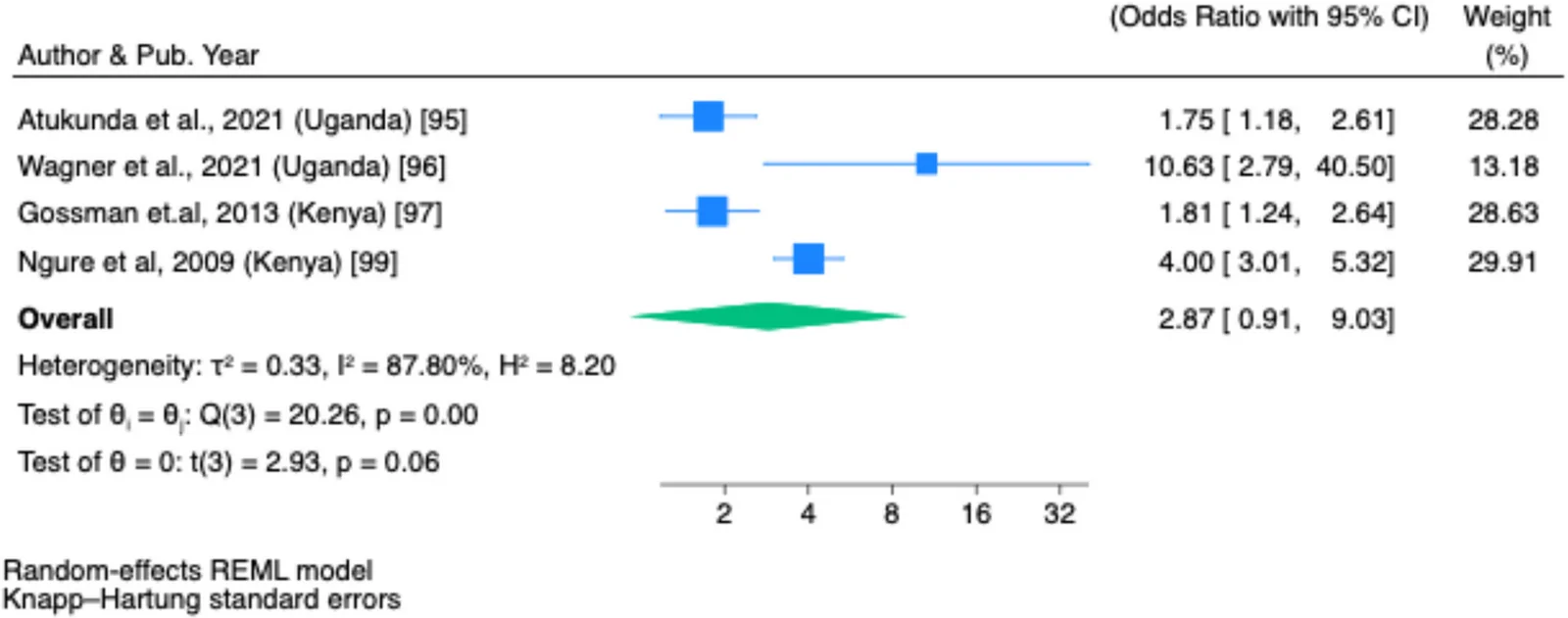
Forest plot of pooled effect estimate of behavioral interventions on contraceptive use in studies conducted in Africa

Among the trials conducted in the African region [95–97, 99], the pooled odds ratio was 2.87 (95% CI:0.91, 9.03), *p =* 0.06 (Fig. 7). The I^2^ index was 87.80%, *p <* 0.001. The moderator (country of study) accounted for 40.21% of the between-study variance, although we found no statistically significance differences, *p =* 0.244.

### Sensitivity analysis

**Fig. 8:**
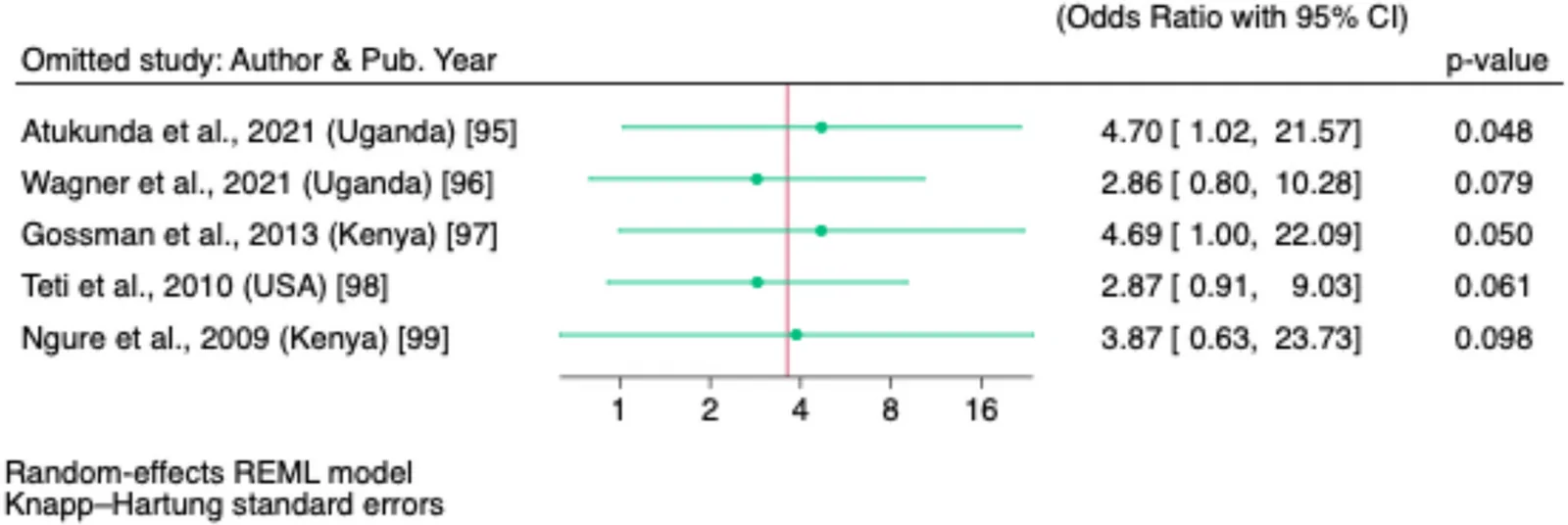
Forest plot of sensitivity analysis

The “leaveoneout” (LOO) sensitivity analysis showed that the pooled odds ratio ranged (OR: 2.86–4.70) (Fig. 8) at each iteration when individual studies were excluded. The direction of effect remained consistent across multiple iterations indicating that no single study unduly influenced the overall effect 3.61 (95% CI: 1.15, 11.32) in our meta-analysis.

### Summary of findings

We adapted the GRADE approach to rate the certainty of the evidence as moderate. The evidence was downgraded by one level for concerns related to risk of bias (allocation concealment, limited blinding and differences in interventions implementation) across some of the studies. The anticipated absolute effect suggests an increase from 974 per 1000 women living with HIV using contraceptives in the control arm to 993 per 1000 in the intervention arm with behavioral interventions (Table 3).

**Table 3:** Summary of Findings: Effect of behavioral interventions compared to usual care on contraceptive use

Summary of findings: Behavioral interventions compared to usual care to increase contraceptive use among women living with HIV						
**Behavioral interventions compared to usual care to increase Contraceptive use among women living with HIV**						
**Patient or population:** Women living with HIV **Setting:** Community, hospital and clinics **Intervention:** Behavioral interventions (Counseling, psychotherapy, motivational interviewing, provision of Family Planning vouchers, cash transfers, text messaging etc.) **Comparison:** usual care						
**Outcomes**	Anticipated absolute effects^*^ (95% CI)		**Relative effect** **(95% CI)**	**№ of participants** **(studies)**	**Certainty of the evidence** **(GRADE)**	**Comments**
	**Risk with usual care**	**Risk with Behavioral interventions**				
Contraceptive use (assessed with: Self-reports)	974 per 1,000	**993 per 1,000** (977 to 998)	**OR 3.6**1 (1.15 to 11.32)	5903 (5 RCTs)	⨁⨁⨁◯ Moderate^a,b,c^	Behavioral interventions likely results in a large increase in contraceptive use
*The risk in the intervention group (and its 95% confidence interval) is based on the assumed risk in the comparison group and the **relative effect** of the intervention (and its 95% CI) **CI:** confidence interval; **OR:** odds ratio						
**GRADE Working Group grades of evidence** **High certainty:** we are very confident that the true effect lies close to that of the estimate of the effect **Moderate certainty:** we are moderately confident in the effect estimate: the true effect is likely to be close to the estimate of the effect, but there is a possibility that it is substantially different **Low certainty:** our confidence in the effect estimate is limited: the true effect may be substantially different from the estimate of the effect **Very low certainty:** we have very little confidence in the effect estimate: the true effect is likely to be substantially different from the estimate of effect						

Explanations

^a^In two studies, it was unclear whether intervention providers and participants were blinded which may have influenced self-reported contraceptive use

^b^Sample size differed largely; inflated effect estimates were generated from smaller studies compared to the larger ones

^c^Intervention implementation differed across the studies (dose and content)

## Discussion

Women living with HIV experience high unplanned pregnancy and contraceptive discontinuation compared to women without HIV [15]. However, contraception among women living with HIV has been low [28, 29], and remains an important public health issue. We examined the effect of behavioral interventions on contraceptive use among women living with HIV using randomized controlled trials. Across the studies reviewed, structured contraceptive counseling interventions focused on family size choices, access to family planning, awareness creation, safe sex, information on HIV and sexually transmitted infections (STIs), condom use negotiation and free provision of contraceptives and vouchers, integration of contraceptive counseling into HIV care were the main interventions [95, 96, 98, 99]. In each of the studies reviewed, the interventions implemented were significantly associated with greater odds of contraceptive use compared to controls [95–99], demonstrating that behavioral interventions may improve contraceptive use among women living with HIV.

Although we may be cautious in interpreting these findings due to the small number of studies and the methodological limitations highlighted, this result nonetheless is in line with a prior systematic review by Lopez and colleagues that reported an overall positive effect of behavioral interventions on contraceptive use among women living with HIV [39]. However, the review by Lopez and colleagues included varied study designs (non-randomized studies) and interventions which limited their ability to statistically examine the effect of behavioral interventions on contraceptive use [39]. Our review specifically builds on this by estimating the pooled effect of behavioral interventions on contraceptive use among women living with HIV across randomized controlled trials (RCTs). However, our findings should be interpreted within the context of the high heterogeneity, potentially arising from variations in intervention components, duration and dose, control conditions, small number of trials included and the use of self-reports which may have led to social desirability biases. These methodological limitations including self-reports of outcomes may have influenced the observed effect of behavioral interventions across the studies included in our review. While the findings may suggest potential benefit of behavioral interventions on contraceptive use among women living with HIV, the evidence is moderate, and further well-designed randomized controlled trials are warranted to validate these findings.

We performed sub-group analysis to evaluate heterogeneity in our review. We noted differences in effect estimates across subgroups compared to the overall finding, although these were not statistically significant. Similarly, heterogeneity remained, reflecting plausible true differences in effect estimates of individual studies, study sizes, and methodological differences or variation in intervention components and how they were designed and implemented [101]. Although, we noted an increased pooled odds ratio across studies that implemented contraceptive counseling compared to the overall effect size, this finding was not statistically significant in this subgroup.

The small number of trials included, presence of heterogeneity and methodological limitations highlighted limit our confidence in the precision and robustness of this finding. The increased effect estimate in this subgroup compared to the overall effect estimate may reflect random variation, small-study effects or underlying clinical and methodlogical variation rather than true effect modification and should be interpreted with caution.

While high rates of unpplanned pregnancy has been reported among women living with HIV, contraceptive use differs among women living with HIV and those without: with women living with HIV reporting lower contraceptive use compared to those without HIV [102]. As with the general population of women, women living with HIV often face significant barriers accessing conraceptives: concerns about drug-drug interactions associated with ART and contraceptive use, stigma, side effects and the need to balance pregnancy prevention with safer conception that could uniquely shape their contraceptive adoption and use [103]. Notably, contraception offers an effective approach to preventing unplanned pregancy, reduce maternal and neonatal mortality and morbidity due to unsafe abortions and vertical HIV transmission [11]. Optimizing behaviorally-informed counseling-based approaches within HIV care through targeted intervention implementation modalities could offer opportunities to adress individual barriers, enhance contraceptive use and reduce unplanned pregnancy among WLHIV. Sustained contraceptive use contituation, ability to switch contraceptive methods on an evolving need and preference, and staisfaction with method choice are critical for achieving long-term reproductive health outcomes for women living with HIV.

Altogether, our meta-analysis demonstrated effect of behavioral interventions on contraception among women living with HIV, suggesting that incorporating behavioral interventions into HIV care may offer an opportunity to enhance contraceptive use, reduce unplanned pregnancy and improve reproductive health outcomes for women living with HIV. Such interventions should support ongoing use, patient-centered counseling and long-term choice. Our evidence is moderate given the methodological limitations and the results should be interpreted cautiosly.

### Strenths and limitations of the review

This review has some strengths and limitations. Firstly, the search strategy was designed in collaboration with an expert medical librarian and conducted across multiple databases. This ensured that a broad literature base was searched to identify and include potentially relevant studies. Similarly, the literature search was not limited to a particular country and publication year, ensuring that relevant studies were identified and increasing the generalizability of the review finding to women living with HIV. Also, the review was conducted following the PRISMA guidelines. However, subgroup analyses were limited and constrained due to the small number of studies included in this review. As a consequence, the pooled effect estimates and tests of subgroup differences may have limited interpretatbility and statistical power. Similarly, we were unable to formally assess publication bias due to the small number of trials included.

Although the limited number of trials may influence the review findings with potential for type 1 error [104, 105], the findings point in similar direction when compared to the individual studies included and a prior study [39]. Another limitation is the inclusion of peer reviewed studies published in English language only. This may have resulted in the exclusion of potentially relevant studies which could have influenced review findings and conclusions. Further, outcome measures were assessed using self reports which could have resulted in social desirability and recall biases [106], and has the potential to overestimate intervention effects in the primary studies included. Additionally, the included studies span 2004–2019, and these may have introduced variability in contextual factors including contraceptive access and delivery across the diverse study settings, and cultutal norms which may have influenced intervention implementation and outcomes. Ultimately, the review findings are to be interpreted against these strentghs and limitations.

### Implications for policy and practice

This review demonstrated the effect of behavioral interventions on contraceptive use and provides important policy and program development insights in reproductive health among women living with HIV. The findings underscore the potential of behaviorally informed approaches which could support informed contraceptive decision-making, and may lead to improved reproductive autonomy, reduce unplanned pregnancy and improve overall reproductive health outcomes for women living with HIV [107–109]. Expanding access to tailored counseling and educational programs, addressing unique psychological, physical and structural barriers represents a strategic approach aimed at increasing contraception among women living with HIV. Behavioral interventions that includes empowerment-based counseling, condom-negotiation skills and individualized reproductive goal setting are therefore critical and warranted. Further, integration of counseling-based approaches into HIV care may offer a holistic approach to care, reduce missed opportunities and ensure that reproductive health is delivered as an essential component of HIV care. In resource-limited settings including SSA, counseling-based approaches may offer a great potential for improving contraceptive use among women living with HIV, reduce the burden of unplanned pregnancy and vertical transmission of HIV.

Because high quality counseling has a potential to positively influence contraceptive use [110], targeted and age-appropriate counseling-based interventions to enhance contraceptive use among women living with HIV are warranted. Similarly, continuous in-service training of healthcare staffs on emerging person-centered contraceptive care and counseling models are encouraged to ensure delivery of high standard contraceptive care that is responsive to the needs of women living with HIV. However, while these behavioral interventions have demonstrated the potential to improve contraceptive use, recommendations for their integration and scale-up within HIV care are to be approached cautiously, taking into consideration, the small number of trials included and methodological limitations highlighted.

### Implications for research

Limited randomized controlled trials were included in this meta-analysis. Therefore, large multicenter trials across more populations particularly in regions with high HIV burden and low contraceptive use are warranted. In addition, none of the studies stratified their data by contraceptive type among women living with HIV. Such stratification would have allowed for evaluating the effect on contraceptive use by method type particularly among women living with HIV in low resource settings. Future studies could consider evaluating these to guide policy and practice. Given our findings on overall effect of behavioral intereventions from short-term trials reviewed, future studies are needed to explore long-term impact of behavioral interventions on contraceptive use and sustainability across diverse populations of women living with HIV. This will help establish whether contraceptive use is maintained over time, particularly when the intervention ends.

Further, studies to examine factors that contributes to long-term use and sustenance are also warranted. Across the studies that implemented multi-component behavioral interventions, none presented disagregated data on the different components. Therefore, additional studies to disentangle the most effective behavioral interventions in improving contraceptive use among women living with HIV are needed. Also, additional implementation science studies evaluating models of contraceptive counseling, family planning integration into HIV care and long term effects on contraceptive use, pregnancy and HIV outcomes are needed. Similarly, cost-effectiveness studies of behavioral interventions on contraceptive use among women living with HIV are encouraged.

## Conclusion

This systematic review and meta-analysis found that overall, behavioral interventions have the potential to improve contraceptive use among women living with HIV. Enhancing contraceptive counseling and expanding access to behavioral interventions could enhance contraception, promote reproductive autonomy, and help women living with HIV achieve their reproductive goals. However, our evidence is limited and considered moderate due to the small number of trials included and methodological limitations highlighted. Therefore, the review findings should be interpreted cautiously, and further well-designed randomized controlled trials are warranted to validate the effect and generalizability of these interventions among women living HIV.

## Supplementary Information

Supplementary Material 1.

## Data Availability

No datasets were generated or analysed during the current study.

## Appendix 1

### Full search strategy

**Table:**

Database	SEARCH STRATEGY
PubMed	(“family planning”[tiab] OR “family planning services”[tiab] OR "Family Planning Services"[Mesh] OR contraception[tiab] OR "Contraception"[Mesh] OR contraceptives[tiab] OR contraceptive[tiab] OR “contraceptive agents”[tiab] OR "Contraceptive Agents"[Mesh] OR “contraceptive devices”[tiab] OR "Contraceptive Devices"[Mesh] OR IUD[tiab] OR “intra-uterine device”[tiab] OR "intrauterine device"[tiab] OR “intra-uterine devices”[tiab] OR “intrauterine devices”[tiab] OR “birth control”[tiab] OR “birth spacing”[tiab] OR “birth intervals”[tiab] OR "Birth Intervals"[Mesh] OR “planned pregnancy”[tiab] OR “planned pregnancies”[tiab]) AND (“Human Immunodeficiency Virus”[tiab] OR HIV[tiab] OR "HIV"[Mesh] OR “Immunodeficiency Virus” [tiab] OR “Immunodeficiency Viruses” [tiab] OR “Acquired Immune Deficiency Syndrome” [tiab] OR “Acquired Immunodeficiency Syndrome” [tiab] OR "Acquired Immunodeficiency Syndrome"[Mesh] OR AIDS[tiab] OR “women living with HIV”[tiab] OR “HIV seropositive”[tiab] OR “HIV Seropositivity”[tiab] OR "HIV Seropositivity"[Mesh] OR “infected with HIV”[tiab] OR “HIV infection”[tiab] OR “HIV infections”[tiab] OR "HIV Infections"[Mesh]) AND (“behavioral interventions”[tiab] OR “counseling”[tiab] OR "Counseling"[Mesh] OR “contraceptive counseling”[tiab] OR “reproductive counseling”[tiab] OR “reproductive counselling”[tiab] OR “sex counseling”[tiab] OR “sex counselling”[tiab] OR “psychotherapy”[tiab] OR "Psychotherapy"[Mesh] OR “psychosocial interventions”[tiab] OR “motivational interviewing”[tiab] OR "community-based family planning"[tiab] OR “family planning vouchers”[tiab] OR “vouchers”[tiab] OR “texting”[tiab] OR “text messaging”[tiab] OR "Text Messaging"[Mesh] OR “mHealth”[tiab] OR “m-Health”[tiab] OR “eHealth”[tiab] OR “e-Health”[tiab] OR “telehealth”[tiab] OR “tele-health”[tiab] OR “tele-medicine”[tiab] OR “telemedicine”[tiab] OR "Telemedicine"[Mesh] OR “tele-counseling”[tiab] OR “telecounseling”[tiab] OR “cash transfers”[tiab] OR “conditional cash transfers” [tiab]) AND ("randomized controlled trial" OR "randomised controlled trial" OR “RCT” OR “randomized trial” OR "Randomized Controlled Trial" [Publication Type] OR "Randomized Controlled Trials as Topic"[Mesh])
Web of Science	(“family planning” OR “family planning services” OR “contraception” OR “contraceptives” OR “contraceptive” OR “contraceptive agents” OR “contraceptive devices” OR IUD OR “intra-uterine device” OR "intrauterine device" OR “intra-uterine devices” OR “intrauterine devices” OR “birth control” OR “birth spacing” OR “birth intervals” OR “planned pregnancy” OR “planned pregnancies”) AND (“Human Immunodeficiency Virus” OR “HIV” OR “Immunodeficiency Virus” OR “Immunodeficiency Viruses” OR “Acquired Immune Deficiency Syndrome” OR “Acquired Immunodeficiency Syndrome” OR “AIDS” OR “women living with HIV” OR “HIV seropositive” OR “HIV Seropositivity” OR “infected with HIV” OR “HIV infection” OR “HIV infections”) AND (“behavioral interventions” OR “counseling” OR “contraceptive counseling” OR “reproductive counseling” OR “reproductive counselling” OR “sex counseling” OR “sex counselling” OR “psychotherapy” OR “psychosocial interventions” OR “motivational interviewing” OR "community-based family planning" OR “family planning vouchers” OR “vouchers” OR “texting” OR “text messaging” OR “mHealth” OR “m-Health” OR “eHealth” OR “e-Health” OR “telehealth” OR “tele-health” OR “tele-medicine” OR “telemedicine” OR “tele-counseling” OR “telecounseling” OR “cash transfers” OR “conditional cash transfers”) AND ("randomized controlled trial" OR "randomised controlled trial" OR “RCT” OR “randomized trial”)
Embase	('family planning':ti,ab,kw OR 'family planning services':ti,ab,kw OR 'family planning'/exp OR 'contraception':ti,ab,kw OR 'contraception'/exp OR 'contraceptives':ti,ab,kw OR 'contraceptive':ti,ab,kw OR 'contraceptive agents':ti,ab,kw OR 'contraceptive agent'/exp OR 'contraceptive devices':ti,ab,kw OR 'contraceptive device'/exp OR 'iud':ti,ab,kw OR 'intra-uterine device':ti,ab,kw OR 'intrauterine device':ti,ab,kw OR 'intra-uterine devices':ti,ab,kw OR 'intrauterine devices':ti,ab,kw OR 'birth control':ti,ab,kw OR 'birth spacing':ti,ab,kw OR 'birth intervals':ti,ab,kw OR 'birth interval'/exp OR 'planned pregnancy':ti,ab,kw OR 'planned pregnancies':ti,ab,kw) AND ('human immunodeficiency virus':ti,ab,kw OR 'hiv':ti,ab,kw OR 'hiv'/exp OR 'human immunodeficiency virus'/exp OR 'immunodeficiency virus':ti,ab,kw OR 'immunodeficiency viruses':ti,ab,kw OR 'acquired immune deficiency syndrome':ti,ab,kw OR 'acquired immune deficiency syndrome'/exp OR 'acquired immunodeficiency syndrome':ti,ab,kw OR 'acquired immunodeficiency syndrome'/exp OR 'aids':ti,ab,kw OR 'aids'/exp OR 'women living with hiv':ti,ab,kw OR 'women living with hiv'/exp OR 'hiv seropositive':ti,ab,kw OR 'hiv seropositivity':ti,ab,kw OR 'hiv seropositivity'/exp OR 'infected with hiv':ti,ab,kw OR 'hiv infection':ti,ab,kw OR 'hiv infection'/exp OR 'hiv infections':ti,ab,kw OR 'hiv infections'/exp OR 'human immunodeficiency virus infection'/exp) AND ('behavioral interventions':ti,ab,kw OR 'counseling':ti,ab,kw OR 'counseling'/exp OR 'contraceptive counseling':ti,ab,kw OR 'contraceptive counseling'/exp OR 'reproductive counseling':ti,ab,kw OR 'reproductive counselling':ti,ab,kw OR 'sex counseling':ti,ab,kw OR 'sex counseling'/exp OR 'sex counselling':ti,ab,kw OR 'psychotherapy':ti,ab,kw OR 'psychotherapy'/exp OR 'psychosocial interventions':ti,ab,kw OR 'motivational interviewing':ti,ab,kw OR 'motivational interviewing'/exp OR 'community-based family planning':ti,ab,kw OR 'family planning vouchers':ti,ab,kw OR 'vouchers':ti,ab,kw OR 'texting':ti,ab,kw OR 'texting'/exp OR 'text messaging':ti,ab,kw OR 'text messaging'/exp OR 'mhealth':ti,ab,kw OR 'mhealth'/exp OR 'm-health':ti,ab,kw OR 'ehealth':ti,ab,kw OR 'ehealth'/exp OR 'e-health':ti,ab,kw OR 'e health'/exp OR 'telehealth':ti,ab,kw OR 'telehealth'/exp OR 'tele-health':ti,ab,kw OR 'tele health'/exp OR 'tele-medicine':ti,ab,kw OR 'tele medicine'/exp OR 'telemedicine':ti,ab,kw OR 'telemedicine'/exp OR 'tele-counseling':ti,ab,kw OR 'tele counseling'/exp OR 'telecounseling':ti,ab,kw OR 'cash transfers':ti,ab,kw OR 'conditional cash transfers':ti,ab,kw) AND ('randomized controlled trial'/exp OR 'randomized controlled trial' OR 'randomized controlled trial (topic)'/exp OR 'randomized controlled trial (topic)' OR 'rct' OR 'randomized trial') AND ('article'/it OR 'article in press'/it)
Cochrane CENTRAL	(“family planning” OR “family planning services” OR “contraception” OR “contraceptives” OR “contraceptive” OR “contraceptive agents” OR “contraceptive devices” OR “IUD” OR “intra-uterine device” OR "intrauterine device" OR “intra-uterine devices” OR “intrauterine devices” OR “birth control” OR “birth spacing” OR “birth intervals” OR “planned pregnancy” OR “planned pregnancies”) AND (“Human Immunodeficiency Virus” OR “HIV” OR “Immunodeficiency Virus” OR “Immunodeficiency Viruses” OR “Acquired Immune Deficiency Syndrome” OR “Acquired Immunodeficiency Syndrome” OR “AIDS” OR “women living with HIV” OR “HIV seropositive” OR “HIV Seropositivity” OR “infected with HIV” OR “HIV infection” OR “HIV infections”) AND (“behavioral interventions” OR “counseling” OR “contraceptive counseling” OR “reproductive counseling” OR “reproductive counselling” OR “sex counseling” OR “sex counselling” OR “psychotherapy” OR “psychosocial interventions” OR “motivational interviewing” OR "community-based family planning" OR “family planning vouchers” OR “vouchers” OR “texting” OR “text messaging” OR “mHealth” OR “m-Health” OR “eHealth” OR “e-Health” OR “telehealth” OR “tele-health” OR “tele-medicine” OR “telemedicine” OR “tele-counseling” OR “telecounseling” OR “cash transfers” OR “conditional cash transfers”) AND ("randomized controlled trial" OR "randomised controlled trial" OR “RCT” OR “randomized trial”)
CINAHL Complete	(“family planning” OR “family planning services” OR “contraception” OR “contraceptives” OR “contraceptive” OR “contraceptive agents” OR “contraceptive devices” OR “IUD” OR “intra-uterine device” OR "intrauterine device" OR “intra-uterine devices” OR “intrauterine devices” OR “birth control” OR “birth spacing” OR “birth intervals” OR “planned pregnancy” OR “planned pregnancies”) AND (“Human Immunodeficiency Virus” OR “HIV” OR “Immunodeficiency Virus” OR “Immunodeficiency Viruses” OR “Acquired Immune Deficiency Syndrome” OR “Acquired Immunodeficiency Syndrome” OR “AIDS” OR “women living with HIV” OR “HIV seropositive” OR “HIV Seropositivity” OR “infected with HIV” OR “HIV infection” OR “HIV infections”) AND (“behavioral interventions” OR “counseling” OR “contraceptive counseling” OR “reproductive counseling” OR “reproductive counselling” OR “sex counseling” OR “sex counselling” OR “psychotherapy” OR “psychosocial interventions” OR “motivational interviewing” OR "community-based family planning" OR “family planning vouchers” OR “vouchers” OR “texting” OR “text messaging” OR “mHealth” OR “m-Health” OR “eHealth” OR “e-Health” OR “telehealth” OR “tele-health” OR “tele-medicine” OR “telemedicine” OR “tele-counseling” OR “telecounseling” OR “cash transfers” OR “conditional cash transfers”) AND ("randomized controlled trial" OR "randomised controlled trial" OR “RCT” OR “randomized trial”)

## Abbreviations

HIV: Human Immunodeficiency Virus
PRISMA: Preferred Reporting Items for Systematic Reviews and Meta-Analysis
REM: Random Effects Model
ART: Anti-Retroviral Therapy
RCT: Randomized Controlled Trials
OR: Odds Ratio
CI: Confidence Interval
HCPs: Health Care Providers
GRADE: Grading of Recommendations, Assessment, Development and Evaluation
